# The environmental and ecological determinants of elevated Ross River Virus exposure in koalas residing in urban coastal landscapes

**DOI:** 10.1038/s41598-021-83919-1

**Published:** 2021-02-24

**Authors:** Brian J. Johnson, Amy Robbins, Narayan Gyawali, Oselyne Ong, Joanne Loader, Amanda K. Murphy, Jon Hanger, Gregor J. Devine

**Affiliations:** 1 Mosquito Control Laboratory, QIMR Berghofer Medical Research Institute, Brisbane, QLD 4006 Australia; 2 Endeavour Veterinary Ecology Pty Ltd, 1695 Pumicestone Rd, Toorbul, QLD 4510 Australia; 3 School of Public Health and Social Work, Queensland University of Technology, Kelvin Grove, QLD 4059 Australia

**Keywords:** Conservation biology, Ecological epidemiology, Animal behaviour, Infectious diseases, Viral infection, Entomology

## Abstract

Koala populations in many areas of Australia have declined sharply in response to habitat loss, disease and the effects of climate change. Koalas may face further morbidity from endemic mosquito-borne viruses, but the impact of such viruses is currently unknown. Few seroprevalence studies in the wild exist and little is known of the determinants of exposure. Here, we exploited a large, spatially and temporally explicit koala survey to define the intensity of Ross River Virus (RRV) exposure in koalas residing in urban coastal environments in southeast Queensland, Australia. We demonstrate that RRV exposure in koalas is much higher (> 80%) than reported in other sero-surveys and that exposure is uniform across the urban coastal landscape. Uniformity in exposure is related to the presence of the major RRV mosquito vector, *Culex annulirostris*, and similarities in animal movement, tree use, and age-dependent increases in exposure risk. Elevated exposure ultimately appears to result from the confinement of remaining coastal koala habitat to the edges of permanent wetlands unsuitable for urban development and which produce large numbers of competent mosquito vectors. The results further illustrate that koalas and other RRV-susceptible vertebrates may serve as useful sentinels of human urban exposure in endemic areas.

## Introduction

The koala (*Phascolarctos cinereus*) is an important emblem of Australia’s biodiversity and a globally-recognised Australian icon, yet many populations continue to decline^1^. This decline is largely attributable to wide-scale habitat loss, much of it unregulated. During the period from 2000 to 2017, over 7.7 million ha of Australia’s wildlife habitat was cleared with less than 10% being subject to scrutiny under the Environment Protection and Biodiversity Conservation Act^2^. Of the land lost, ca. 1 million ha was known or potential koala habitat. Such significant habitat losses and associated population declines have resulted in the koala being listed as vulnerable or extinct in much of its historic range^3^. There are pockets where the koala is thriving, but these areas represent a fraction of its native range^4,5^. In the state of Queensland, the koala is currently listed as vulnerable in response to ongoing habitat loss and the threats of climate change^1,6,7^. The historical clearing of primary eucalypt forest habitat and accelerated coastal development in the southeast of the state has been associated with particularly sharp population declines^8–10^.

Habitat loss and climate change are not the only threats to the survival of the koala. Infectious disease has also contributed to its decline. The bacterium *Chlamydia pecorum* causes debilitating ocular and urogenital tract disease while the koala retrovirus (KoRV) has been implicated in host immunosuppression and exacerbation of chlamydial pathogenesis^11–14^. Koalas are also known to be infected by endemic mosquito-borne viruses such Barmah Forest virus (BFV) and Ross River virus (RRV)^15,16^, yet the threat of these viruses to koala health remains poorly studied. RRV is Australia’s most medically important mosquito-borne virus that causes debilitating polyarthritis in humans, among other symptoms^17^. The pathology of RRV in marsupial hosts is currently unknown although myositis and arthritis are reported in mice^18^ and domestic horses^19^. Primary vertebrate hosts are often assumed to be native macropods (e.g. wallabies and kangaroos), but there is considerable debate as to which animals are the primary hosts in urban landscapes where macropods are uncommon but where spillover to humans is often pronounced^20,21^. Although koalas can be locally abundant in the urban landscape as long as suitable green space exists, their relative scarcity and irregular distribution in that environment makes them an unlikely amplifying host for RRV. Remnant urban green spaces are also often resource-poor and therefore may compromise the immunological fitness of koalas living in such spaces^22,23^. Consequently, where RRV exposure is common in urban landscapes, koalas may incur substantial morbidity. Although surveys of captive koalas in New South Wales, Australia, did not find RRV antibodies (n = 12)^24^, surveys performed in southern Victoria, Australia, detected RRV antibodies in 16% of wild koalas (n = 93)^15,16^. This latter observation suggests RRV exposure in koalas may present a health risk, but the literature is sparse and exposure among wild koala populations remains largely unknown.

Here, we performed a large, spatially and temporally explicit RRV seroprevalence survey of koalas residing in the urban coastal landscape using sera collected as part of a broader population health survey performed in response to regional infrastructure development. Our purpose was to examine patterns of RRV exposure in coastal koala populations and identify potential correlates of risk related to koala movement and habitat use. Surveys were focused in urban coastal estuarine and lacustrine environments proximate to human developments. These environments are thought to be important sources of RRV spill over to humans^25,26^. Characterising the prevalence of RRV exposure in koalas in these environments might inform a better understanding of RRV transmission from both a public health and a wildlife conservation perspective (Table 1).

**Table 1:** Summary of major and minor water body classes present in each surveyed environment.

	Major water class	Minor water class	Other water classes
Estuarine	Tidally influenced estuarine: characterised by mangroves and related tree communities	Freshwater palustrine: coastal/ sub-coastal: floodplain tree swamps (melaleuca and eucalypt)	Freshwater lacustrine: artificial/ highly modified wetlands (dams, ring tanks, irrigation channel)
Lacustrine	Freshwater lacustrine: artificial/ highly modified wetlands (dams, ring tanks, irrigation channel)	Tidally influenced estuarine: characterised by mangroves and related tree communities	Freshwater palustrine: coastal/ sub-coastal: floodplain tree swamps (melaleuca and eucalypt)

## Results

### RRV seroprevalence

**Figure 1:**
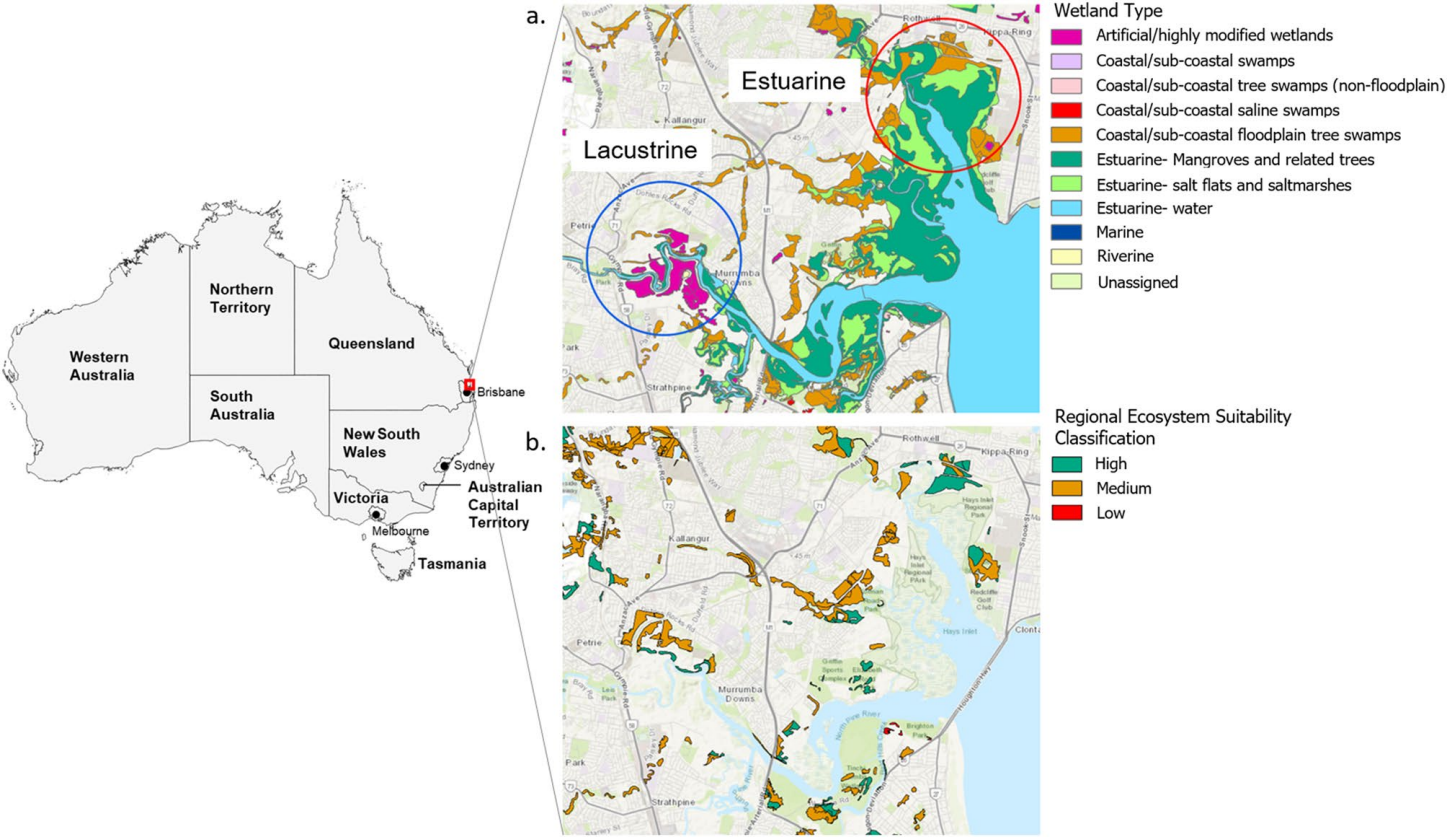
(**a**) Location of the study area in southeast Queensland, Australia, and summary of primary water bodies present in each surveyed environment. (**b**) Location and ecosystem suitability classification of koala habitat in the study area as of the most recent state-wide classification (January 2020)^7^. Wetland^70^ and koala habitat^71^ GIS shapefiles were obtained through the QSpatial Catalogue (http://qldspatial.information.qld.gov.au/). Base layer of the region area sourced from Esri World Imagery^72^. This figure was created using the ArcGIS Pro software suite (version 2.4.2, https://www.esri.com/en-us/arcgis/products/arcgis-pro).

More than 80% of koalas tested in the lacustrine and estuarine habitats surveyed had anti-RRV antibodies. Out of 218 koalas tested, 172 koalas were seropositive, 35 were seronegative, and 11 seroconverted from negative to positive over the course of the study (2015 to 2017). The low number of seroconversions during the study reflects the high prevalence of RRV exposure in each population prior to the beginning of the survey. Seroprevalence rates in female and male koalas were similar (84%, n = 95 vs. 82%, n = 61; *χ*^2^ = 0.07, *p* = 0.80) and not influenced by environment (Table 2). This latter analysis contained data from 187 koalas after accounting for relocated individuals and animals with too few position locations (n < 5) to confirm their assignment to a particular environment. Overall seroprevalence in the estuarine and lacustrine populations surveyed was 82% (n = 73) and 85% (n = 83), respectively. Multivariate logistic regression analyses further revealed that seroprevalence increased with increasing koala age (Table 3), and that these increases were independent of koala sex (male vs female, OR = 1.1, 95% CI 0.5–2.7, *p* = 0.83) and the environment in which they resided (lacustrine vs estuarine, OR = 1.0, 95% CI 0.43–2.4, *p* = 0.98). Koalas aged between 2–4 years, 4–6 years, and > 6 years of age were 6.9 (95% CI 2.5–22.2, *p* < 0.001), 8.2 (95% 2.6–37.2, *p* = 0.001), and 23.7 (4.5–439.9, *p* = 0.003) times more likely to test RRV positive, respectively, relative to the baseline age group (< 2 years of age) (Fig. 1).

**Table 2:** Seroprevalence of Ross River virus (RRV) in koalas residing in urban coastal lacustrine and estuarine environments.

Location	Sample	RRV positive^a^	RRV negative	% positive	Odds-ratios	2.5% CI	97.5% CI	z-value	*p*-value
Estuarine	Overall	73	16	82.02	0.82	0.39	1.82	0.49	0.62
	Female	41	9	82.00	0.99	0.36	2.98	0.01	0.99
	Male	32	7	82.05	1.01	0.34	2.76	0.01	0.99
Lacustrine	Overall	83	15	84.69	1.21	0.55	2.57	0.49	0.62
	Female	54	9	85.71	1.24	0.38	3.88	0.38	0.71
	Male	29	6	82.86	0.81	0.26	2.61	0.38	0.71

Comparison of seroprevalence rates between environments was performed using data only for animals for which their location could be confirmed (n = 187) after accounting for relocated individuals and animals with too few position locations (n < 5) to confirm their assignment to a particular environment.

^a^An animal sampled multiple times was considered seropositive if the animal returned a positive test result during any individual sampling event.

**Table 3:** Summary of seroprevalence results by koala age group and logistic regression outputs for the analysis of the association of koala age with RRV seroprevalence in koalas residing in coastal lacustrine and estuarine environments.

Variable	Seropositive (%)	Seronegative (%)	Seroconverted (%)	Odds ratio (OR)	OR 95% CI	*P-*value
**Age**						
< 2	31 (56.4%)	22 (38.6%)	2 (3.5%)	Baseline	–	–
2–4	48 (85.7%)	5 (8.5%)	3 (5.1%)	6.9	2.5–22.2	< 0.001
4–6	33 (82.5%)	3 (6.8%)	4 (9.1%)	8.2	2.6–37.2	0.001
> 6	33 (91.7%)	1 (2.6%)	2 (5.3%)	23.7	4.5–439.9	0.003

Percentages are calculated for the number of individuals in each row. The data shown is only for animals for which their location could be confirmed (n = 187) after accounting for relocated individuals and animals with too few position locations (n < 5) to confirm their assignment to a particular environment.

### Koala tracking and home range comparisons

**Figure 2:**
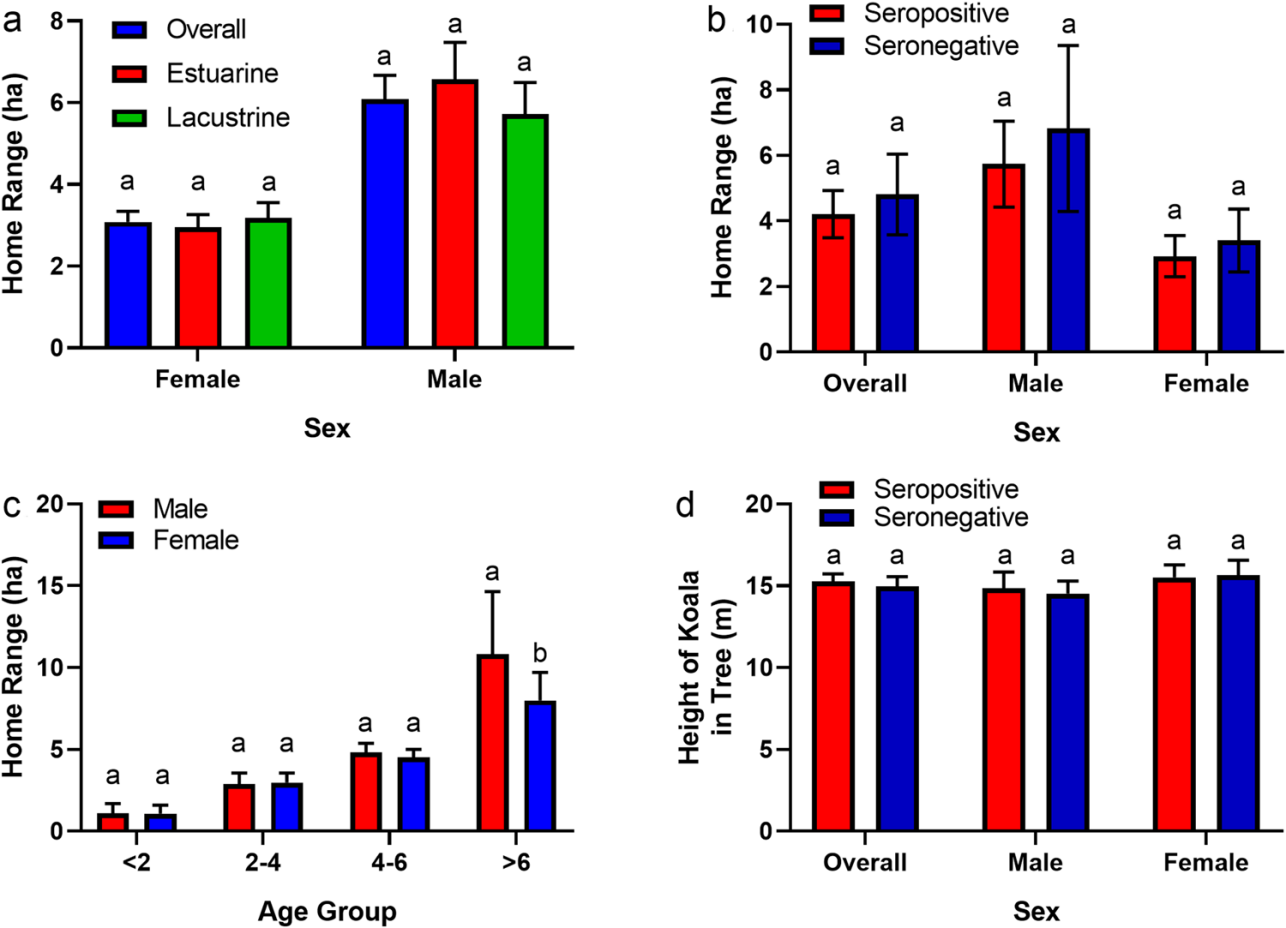
(**a**) Comparison of annual male and female home ranges (mean ± SE), (**b**) comparison of annual home ranges (mean ± SE) of seropositive and seronegative koalas, (**c**) comparison of male and female home ranges by age group (mean ± SE), and (**d**) comparison of koala height (mean ± SE) in trees for seropositive and seronegative koalas. Bars within a graph that do not share similar letters denote statistical significance.

Home ranges were calculated for a total of 155 koalas across the lacustrine (54 females and 37 males) and estuarine (36 females and 28 males) environments after accounting for translocated koalas, those with inadequate sampling duration and those whose home ranges failed to satisfy the Minimum Convex Polygon (MCP) 60% cutoff during visual assessment of home range asymptotes. Home ranges did not differ by environment but did differ by sex (Fig. 2a; *F*_*1, 151*_ = 24.79, *p* < 0.001) with males (mean = 6.1 ha, 95% CI 4.9–7.3 ha) having greater home ranges than females (mean = 3.0 ha, 95% CI 2.6–3.6 ha). However, no significant differences in home ranges was observed within a sex when considering serostatus (Fig. 2b; *F*_1,151_ = 1.57, *p* = 0.21). When home ranges were compared between age groups, males and females exhibited a similar trend of increasing home range size with increasing age (Fig. 2c). Significant differences (*F*_*1,147*_ = 6.28, *p* = 0.01) between males and females were only observed within the oldest age group, wherein male home ranges (mean = 10.8 ha, 95% CI 9.3–12.4 ha) were 26% larger than those of similarly aged females (mean = 8.0 ha, 95% CI 7.0–9.0 ha). Differences between the sexes within the older age group may be attributable to the greater movement of older, dominant males during the mating season^27^. The home ranges of individuals that seroconverted during the study were similar (mean = 6.9 ha, 95% CI 3.4–10.4 ha) to those that were seropositive prior to the study or those that remained seronegative, but too few seroconverted to confidently compare the three groups.

### Koala tree use by environment

Koalas were found to utilize 38 and 56 individual tree species in the estuarine and lacustrine environments, respectively. Although the overall tree communities were unique in relation to the environment, the dominant species used by koalas in each environment were similar (Table 4). Koalas favoured *Eucalyptus tereticornis* (blue gum) and *E. siderophloia* (grey ironbark) in the estuarine environment and *E. racemosa* (scribbly gum) and *E. tereticornis* in the lacustrine environment. Other species commonly used by koalas included *Corymbia intermedia* (pink bloodwood) and *Melaleuca quinquenervia* (broad-leaved paperbark) in the estuarine environment and *Cinnamomum camphora* (camphor laurel) and *C. intermedia* in the lacustrine environment. Tree selection between seropositive and seronegative koalas was similar with nine of the top ten most used tree species being shared between them (Table 5). Both male and female koalas preferred mature trees with greater diameter at breast height (DBH; male 95% CI 40.94–42.96 cm; female 95% CI 42.18–44.62 cm) and occupied similar heights in trees (Fig. 2d; male 95% CI 14.82–15.55 m; female 95% CI 14.09–15.31 m). No significant (*F*_1, 305_ = 0.24, *p* = 0.62) differences for either variable were observed between environments or by serostatus.

**Table 4:** Dominant tree species and characteristics of trees in which koalas were located during the study.

Tree species	Estuarine				Tree species	Lacustrine			
	Count	DBH (cm)	Tree height (m)	Koala height (m)		Count	DBH (cm)	Tree height (m)	Koala height (m)
*Eucalyptus tereticornis* (blue gum)	6446	53.62	21.07	16.61	*Eucalyptus racemosa* (scribbly gum)	2268	66.79	22.08	18.38
*Eucalyptus siderophloia* (grey ironbark)	3937	40.30	19.27	16.10	*Eucalyptus tereticornis* (blue gum)	2157	62.02	21.39	16.36
*Melaleuca quinquenervia* (paperbark)	882	25.73	13.79	12.01	*Cinnamomum camphora* (camphor laurel)	1145	45.39	15.12	12.89
*Corymbia intermedia* (pink bloodwood)	835	39.65	18.45	15.33	*Eucalyptus grandis* (flooded gum)	953	46.02	26.25	21.71
*Casuarina glauca* (she-oak)	738	25.71	13.87	11.67	*Corymbia intermedia* (pink bloodwood)	885	36.72	18.39	14.58
*Corymbia tessellaris* (Moreton Bay ash)	226	40.99	19.59	15.26	*Lophostemon confertus* (brushbox)	586	40.16	19.22	16.89
*Lophostemon confertus* (brushbox)	101	39.03	19.39	16.74	*Eucalyptus siderophloia* (grey ironbark)	398	38.74	19.33	15.61
Mangrove	93	15.72	7.36	6.25	*Melaleuca quinquenervia* (paperbark)	373	34.77	14.07	12.11
*Eucalyptus grandis* (flooded gum)	90	80.70	21.74	16.29	*Casuarina glauca* (she-oak)	276	27.02	13.00	10.56
*Lophostemon suaveolens* (swampbox)	70	25.06	11.71	9.91	*Eucalyptus pilularis* (blackbutt)	228	77.66	29.53	24.44
*Acacia sp.*	50	18.04	9.58	7.65	*Lophostemon suaveolens* (swampbox)	181	23.75	13.57	11.10
*Eucalyptus sp.*	45	52.29	17.64	14.07	*Pinus elliottii* (slash pine)	174	31.88	18.93	15.90
*Corymbia citriodora* (spotted gum)	33	58.79	22.00	18.88	*Ficus sp.*	170	60.20	14.49	11.94
*Eucalyptus propinqua* (grey gum)	29	37.21	14.38	11.92	*Eucalyptus propinqua* (grey gum)	143	49.21	17.61	14.98
Dead tree	12	23.08	10.92	9.42	*Acacia sp.*	134	25.82	11.55	8.45
*Eucalyptus fibrosa* (broad-leaved ironbark)	12	40.42	20.00	17.00	*Corymbia tessellaris* (Moreton Bay ash)	126	36.10	18.87	16.97
*Eucalyptus robusta* (swamp mahogany)	12	41.83	14.67	12.50	*Eucalyptus microcorys* (tallowwood)	126	51.94	20.17	15.53
*Eucalyptus microcorys* (tallowwood)	11	44.27	17.27	12.38	*Pinus sp.*	63	34.57	18.14	15.75
*Ficus sp.*	9	44.89	10.11	7.33	Mangrove	59	27.81	11.58	11.25

Count represents the total number of observations (animal relocations) during which a koala was observed on an individual tree species. The data presented for tree diameter at breast height (DBH), tree height, and koala height in trees represents the mean data for all koalas surveyed in each environment. The tree species shown represents > 95% of animal relocations in each environment.

**Table 5:** Common tree species used by Ross River virus seropositive and seronegative koalas across both study areas.

Seropositive Koalas			Seronegative Koalas		
Tree species	% of animal relocations	# of relocations	Tree species	% of animal relocations	# of relocations
*Eucalyptus tereticornis* (blue gum)	27.51	6799	*Eucalyptus tereticornis* (blue gum)	38.61	2218
*Eucalyptus siderophloia* (grey ironbark)	13.27	3279	*Eucalyptus siderophloia* (grey ironbark)	23.32	1340
*Eucalyptus racemosa* (scribbly gum)	8.66	2141	*Corymbia intermedia* (pink bloodwood)	7.42	426
*Corymbia intermedia* (pink bloodwood)	5.73	1417	*Melaleuca quinquenervia* (paperbark)	6.18	355
*Cinnamomum camphora* (camphor laurel)	4.56	1127	*Casuarina glauca* (she-oak)	4.09	235
*Eucalyptus grandis* (flooded gum)	4.16	1028	*Eucalyptus moluccana* (gum-topped box)	2.68	154
*Melaleuca quinquenervia* (paperbark)	4.05	1002	*Eucalyptus racemosa* (scribbly gum)	2.30	132
*Eucalyptus moluccana* (gum-topped box)	3.69	911	*Corymbia citriodora* (spotted gum)	2.11	121
*Casuarina glauca* (she-oak)	3.38	835	*Lophostemon confertus* (brushbox)	2.00	115
*Lophostemon confertus* (brushbox)	2.64	652	*Lophostemon suaveolens* (swampbox)	1.38	79

Tree use is represented by the percentage (mean) of animal relocations during which a koala was observed on an individual tree species.

### Mosquito community composition

As expected, the estuarine environment contained more of the saltmarsh associated species *Aedes vigilax*, *Culex sitiens* and *Aedes alternans* relative to the lacustrine environment that harbored a greater diversity and abundance of freshwater associated species such as *Culex annulirostris* and *Aedes procax* (Table 6). *Cx. annulirostris* dominated both habitats, accounting for 86% and 41% of all collections from the lacustrine and estuarine environments, respectively. While the abundance of mosquitoes in both environments was substantial, the estuarine environment had the greatest number of mosquitoes per surveillance event (11,199 ± 2,688 vs. 5,576 ± 2,696).

**Table 6:** Summary of mosquito species collected in each surveyed environment.

	Total collected	Mean	Std. error of mean	% of total collected (mean)	SE of % of total
**Lacustrine**					
*Aedes alternans*	0	0	0	0	0
*Aedes aculeatus*	33	11	8.62	0.77	0.59
*Aedes notoscriptus*	202	67.33	37.95	1.79	0.68
*Aedes procax*	139	139	0	0.62	0.62
*Aedes vigilax*	302	100.7	51.36	2.07	0.35
*Aedes vittiger*	0	0	0	0	0
*Anopheles annulipes*	512	170.7	167.7	2.64	1.63
*Culex annulirostris*	13,993	4664	2219	86.25	6.34
*Culex hilli*	0	0	0	0	0
*Culex sitiens*	1518	506	506	5.61	5.61
*Uranotaenia sp.*	29	9.667	9.17	0.25	0.13
**Estuarine**					
*Aedes alternans*	1252	417.3	201.5	3.7	1.19
*Aedes aculeatus*	0	0	0	0	0
*Aedes notoscriptus*	0	0	0	0	0
*Aedes procax*	0	0	0	0	0
*Aedes vigilax*	6755	2252	1068	27.78	17.88
*Aedes vittiger*	50	0	0	0.13	0.13
*Anopheles annulipes*	0	0	0	0	0
*Culex annulirostris*	16,846	8423	80	40.6	20.35
*Culex hilli*	306	153	52	0.73	0.41
*Culex sitiens*	8389	2796	762.9	27.07	6.51
*Uranotaenia sp.*	0	0	0	0	0

Two CO_2_-baited light traps were set at each sampling site across four (n = 4) surveillance events.

## Discussion

The risk of arbovirus transmission to both humans and wildlife is influenced by the environment and is therefore spatially heterogeneous. Distinct patterns of increased human RRV risk in southeast Queensland (SEQ) appear to be driven by environmental changes in vector-vertebrate communities^21,28^, but little is known of the factors driving environmental and spatial risks of RRV transmission to endemic wildlife. In the present study, we demonstrate that RRV seropositivity in koalas can be far greater than that reported for other marsupial species^17,29^ and other circulating arboviruses such as BFV^15,30^. Uniformity in exposure is related to the presence of the major RRV mosquito vector, *Culex annulirostris*, and similarities in koala movement, tree use, and age-dependent increases in exposure risk. The findings suggest that elevated RRV exposure in coastal koala populations is related to the confinement of remaining koala habitat to the edges of permanent wetlands that are unsuitable for further development and which produce large numbers of mosquito vectors. The discovery of high RRV prevalence in these populations supports the need for future investigations into the population health consequences of RRV and other endemic arboviruses in koalas.

Habitat loss and fragmentation disrupts many important ecological processes including population dynamics and resource use^31–33^. It is further linked to increases in human disease risk for a variety of zoonotic diseases within urban environments through processes related to population isolation, reductions in host species richness, and increases in the abundance of urban adapted, highly competent host and vector species^34–37^. The current study reveals, for the first time, high RRV seroprevalence in koala populations residing in degraded urban coastal habitats. Resource related reductions in immunological fitness^22,23^, combined with often elevated animal densities in remnant coastal habitat patches^7^, may contribute to elevated RRV and other infectious disease prevalence in the surveyed populations (35% *C. pecorum,* 100% KoRV-A and 24% KoRV-B)^38^. The influence of habitat disturbance on RRV risk to koalas is further supported by lower seroprevalence rates (13%; n = 93) in koalas residing in the coastal Gippsland Lakes region of Victoria, Australia^15,16^, a region containing ca. 20,000 ha of protected natural habitat. However, we cannot attribute these differences to quality of habitat alone, as climate variability between regions will have differing impacts on RRV transmission intensity. Of note, although tree use by koalas in this study was similar to that reported elsewhere^39–41^, recent infrastructure developments in the study area have resulted in the loss of ca. 53 ha of koala habitat and the removal of at least 17,000 maturing trees^42,43^. This has clearly degraded the quality of this habitat overall and may influence future patterns of koala tree use and resource-related associations with arbovirus exposure in the areas surveyed.

The risk of disease exposure in wildlife often increases with animal age. Age-related increases in seropositivity has been demonstrated in koalas for KoRV^44^, as older animals are more likely to become infected due to having more contact with other koalas. The likelihood of vector-host contact also increases with age and reports on humans^17^ and other marsupial species^24,29^ demonstrate that antibody prevalence often increases with increasing age. Our data reflects the same pattern and is supported by the majority of seronegative koalas being < 2 years of age and the relatively young age of individuals that positively seroconverted during the study. It is difficult to give much weight to the latter finding, however, considering the low number of seroconversions observed. Our results further suggest that exposure is related to age-dependent patterns of movement, as both exposure and movement increased proportionally with increasing koala age. The home ranges observed in this study (males = 6.1 ha, females = 3.3 ha) are similar to those of koalas residing in another urban coastal habitat in Victoria, Australia (males = 9.1 ha, females = 4.3 ha)^45^, and general patterns of movement observed may reflect a combination of age and resource-limited^27,45,46^ effects. Exposure ultimately appears to be dependent upon multiple demographic and habitat related factors, and the interactions between them. Thus, additional studies performed across varied habitats are needed to better understand how such factors influence exposure risk in koalas to RRV and other arboviruses.

Although several mosquito species captured in each environment are known to transmit RRV, including *Cx. annulirostris*, *Ae. vigilax* and *Cx. sitiens*, historical evidence suggests *Cx. annulirostris* is the most likely vector for koalas. This mosquito has a tendency to seek hosts above ground level^47,48^, and commonly blood-feeds on marsupials, including tree-dwelling species like the brushtail possum^49,50^. The presence of *Cx. annulirostris* may expose koalas to other circulating arboviruses, as it is also a principal vector of BFV, Murray Valley encephalitis and West Nile Kunjin viruses^49,51^. Of these, BFV is the second most medically important endemic mosquito-borne disease in SEQ and serological investigations implicate marsupials as the largest and most diverse group of reservoirs^52^. Although previous reports suggest exposure to BFV in koalas is low (9% seroprevalence)^15^, the limited number of studies justifies additional investigations.

Lastly, infection in vertebrate hosts at the wildlife-human interface may be used to forecast the risk of spillover to the human population^53,54^. Although the spatial distribution of human RRV cases is extensive, there are elevated risks associated with suburban areas harbouring a greater proportion of wetlands and bushland and an increased presence of *Cx. annulirostris* and other freshwater mosquitoes^25,28,55^. These observations are corroborated by reports of RRV outbreaks being preceded by above-average rainfall events^56,57^ that provide temporary freshwater larval habitats for *Cx. annulirostris* populations for extended periods of time. Increased monitoring of disease exposure in non-human vertebrate populations, such as koalas, may enhance our understanding of the environmental and ecological determinants of human exposure.

## Conclusion

This survey represents one of the largest single-species marsupial seroprevalence surveys performed to date. The scale and scope of the survey provided key insights into the environmental and ecological determinants of RRV exposure in koalas residing in the urban coastal landscape. Substantial risks of exposure are associated with confinement of remnant coastal koala habitat to the edges of permanent wetland features that produce large numbers of competent mosquito vectors. Particular importance is attributed to the presence of freshwater habitat suitable to *Cx. annulirostris* mosquitoes. These results demonstrate that the careful study and sampling of wildlife populations can yield insights relevant to animal conservation and public health in rapidly changing urban landscapes.

## Methods

### Study area

Koalas were surveyed in coastal estuarine and lacustrine environments (Fig. 1a, Table 1) located in the Moreton Bay Region (MBR) of SEQ (27.2337° S, 153.0683° E and 27.2685° S, 152.9896° E, respectively), Australia. Koala habitat loss and fragmentation due to urban development in the two study areas has been severe over the last decade and remaining koala habitat is largely confined to the edges of swampy and/or flood-prone areas unsuitable for further development (Fig. 1b)^43^. Much of the remaining habitat in the more developed lacustrine study area is heavily fragmented and classified as medium quality, or suitability, for koalas^7^, whereas the less developed estuarine environment contains a greater proportion of high quality habitat. Both surveyed environments harbor mosquito faunas typical of the region’s saltwater (*Ae. vigilax*) and freshwater (*Cx. annulirostris*) habitats because of their proximity to permanent water features. These species are significant vectors of RRV and readily blood-feed on humans and a variety of non-human vertebrates including marsupials^50,58,59^.

Endemic human circulation of RRV occurs in the region, with an average of 259 human cases/year notified between January 2012 and December 2016 (Data provided by the Queensland Department of Health, Communicable Diseases Branch, QIMRB Human Research Ethics Committee Approval no. P2238; Fig. S1). Human disease cases are reported in the region (and in broader SEQ) year-round, commonly peaking between February and May each year. This coincides with the late austral summer and autumn seasons, when seasonal increases in mosquito abundance occur.

### Koala sampling, collaring and aging

We surveyed 223 koalas as part of a regional koala management program undertaken in response to a large, multi-year transport infrastructure development (Moreton Bay Rail Project; see Hanger et al.^43^) that spanned both study areas. Koalas were captured using traps or the flag and pole technique and transported to the Endeavour Veterinary Ecology facilities in Toorbul, SEQ. Here, experienced veterinarians conducted a comprehensive clinical examination under anaesthesia to assess koala health which included the collection of a blood sample from the cephalic vein. During this examination, an estimated year of birth was determined for each koala based on the wear of the upper premolar and molar teeth^60^. For joeys, a date of birth was determined based on the developmental characteristics and size of the joey relative to a reference chart of known-age joeys. Initial age estimation enabled us to determine the approximate age of each koala during subsequent serum collection events. Following examination, healthy koalas were fitted with identification and telemetry devices to facilitate monitoring in the field. After recovery from anaesthesia, they were transported back to the study area for release at their point of capture (or as close as practicable based on safety or welfare concerns). Collaring and tracking of koalas began in March 2013 and ceased in January 2017.

### Koala tracking

Koala tracking and monitoring followed the methods of Robbins et al.^61^ Briefly, koalas weighing more than 3 kg were monitored using near-real-time biotelemetry devices (*K-Tracker* telemetry system, LX Group, Sydney, New South Wales) that reported global positioning system (GPS) locations and activity levels from each tagged koala via 12-hourly data uploads to an internet-based server. Koala position and activity levels were monitored remotely via the internet every 24 h but koalas were also field-tracked using very high frequency (VHF) radiotelemetry at least once per fortnight. Koalas weighing between 1 and 3 kg were not large enough to be fitted with the *K-Tracker* collars, so were field-tracked using VHF radiotelemetry several times a week. Koalas were monitored more frequently if there were health or welfare concerns, or if activity data reported by *K-Tracker* collars indicated low or zero activity. At each field monitoring event, koalas were examined with binoculars and various data recorded, including GPS location, tree DBH, tree species, tree height, the koala’s height in the tree, external signs of health and the presence or absence of joeys. DBH was measured using a DBH tape and the maximum DBH measurement was recorded for each tree. Tree height and koala height was estimated by experienced field technicians. Koalas included in this study were monitored for a period ranging from 1 to 46 months (mean = 7.98; 95% CI = 7.54 to 8.54 months), which enabled determination of movement patterns and resource preferences.

### Home range determination

The geo-referenced position data were used to calculate the home range sizes for each individual koala using the adehabitatHR package in R^62^. Prior to analysis, each position was mapped with QGIS software^63^ to provide a visual indication of home ranges and ensure quality of data. Home range sizes were determined by minimum convex polygon (MCP 95%) estimation for each year the animal was monitored. Mean home range sizes were determined by averaging home range estimates across all surveyed years for each individual. In select cases, annual home ranges were estimated with lower confidence (cutoff MCP ≥ 60%) based on visual inspections of home range asymptote plots.

### Serum collection and determination of seroprevalence

Koala sera were collected opportunistically between 2015 and 2017. A total of 529 blood samples were taken from 218 koalas. Some koalas were bled more than once (range = 1–6 serum samples/koala; mean = 2.3), creating the opportunity to investigate seroconversion in koalas. The presence of neutralising antibodies against RRV in each serum sample was tested in duplicate by preparing a monolayer of mammalian cell lines (Vero) in a 12-well tissue culture plate. Dilutions (1:10) of koala sera in RPMI-1640 (Sigma-Aldrich, USA) and the virus isolate RRV T48^64^ were introduced to each well, sufficient to produce 50 to 60 plaques in the absence of antibody. Plates were incubated for two hours after which the virus-serum mixture was supplemented with 0.75% carboxymethylcellulose (CMC, Sigma-Aldrich, USA) overlay medium in ds-RPMI. Plates were incubated at 37 °C and 5% CO_2_ for a further 2 days, fixed with crystal violet and examined for cytopathic effects (CPE), which can be determined by counting the number of plaques. Neutralising antibodies bind to the virus, preventing CPE and the formation of plaques. Koala sera that neutralised ≥ 50% of plaques in these assays were considered anti-RRV seropositive^65^. This methodology has been deployed in a number of studies^66,67^, and has been tested at dilution rates of > 160 fold confirming strong and specific binding by the antibody^67^. We considered an animal sampled multiple times as seropositive if the animal returned a positive test result during any individual sampling event.

### Mosquito community composition

The mosquito community in each location was characterized from February to March 2020; months associated with high mosquito and RRV activity in the region^68^. Mosquitoes were collected using CDC light traps (Pacific Biologics, Brisbane, Australia) baited with CO_2_ from dry ice on 4 dates. Two traps were set within each environment either weekly or fortnightly depending on weather. Traps were operated from 18:00 to 06:00 during each surveillance event. Captured mosquitoes were transported back to the laboratory where they were stored at − 20 °C until identified to species using standard taxonomic keys.

### Statistical analysis

Fisher’s exact test was used to compare differences in the number of positive sera results obtained in each surveyed environment. Two-way ANOVA was used to compare differences in home range sizes and the mean height of koalas in trees between seropositive and seronegative individuals. We used a generalized linear logistic model with the logit link function to determine the association between sero status and koala age. For this analysis, we grouped koalas into four age groups. Age groups included koalas < 2 years of age, those between 2 and 4 years of age, those between 4 and 6 years of age, and those > 6 years of age. Koala sex and location were included as co-variables in the model.

### Regulatory approvals

The koala management program was conducted under approvals issued by the Queensland Department of Agriculture and Fisheries (approvals CA 2012/03/597, CA 2013/09/719, CA 2014/06/777, CA 2015/03/852, and CA 2016/03/950). Animal ethics and research work was authorized by scientific purposes permits issued by the Queensland Department of Environment and Heritage Protection (approvals WISP 11525212, WISP 16125415, WISP 13661313, WITK 14173714 and WISP 17273716). All experiments were performed in accordance with the relevant guidelines and regulations. All studies involving animals are reported in accordance with the ARRIVE guidelines for reporting experiments involving animals^69^.

## Supplementary Information

Supplementary Information
