# Supplementary material for: The environmental and ecological determinants of elevated Ross River Virus exposure in koalas residing in urban coastal landscapes
Source: Sci Rep. 2021 Feb 24;11:4419. doi: 10.1038/s41598-021-83919-1 (PMC7904799; doi:10.1038/s41598-021-83919-1)
Supplement: Supplementary file 1 — Supplementary Information [file 41598_2021_83919_MOESM1_ESM.docx]

**The environmental and ecological determinants of elevated Ross River Virus exposure in koalas residing in urban coastal landscapes**

Brian J Johnson^1,*, ⸸^, Amy Robbins^2, ⸸^, Narayan Gyawali^1^, Oselyne Ong^1^, Jo Loader^2^, Amanda K. Murphy^1,3^, Jon Hanger^2^, and Gregor J Devine^1^

^1^Mosquito Control Laboratory, QIMR Berghofer Medical Research Institute, Brisbane, QLD 4006, Australia

^2^Endeavour Veterinary Ecology Pty Ltd, 1695 Pumicestone Rd, Toorbul, QLD 4510, Australia

^3^Environmental Epidemiology Group, School of Public Health and Social Work, Queensland University of Technology, Kelvin Grove, QLD 4059

*corresponding author: brian.johnson@qimrberghofer.edu.au

^⸸^These authors contributed equally to this work

**SUPPLEMENTARY MATERIAL**


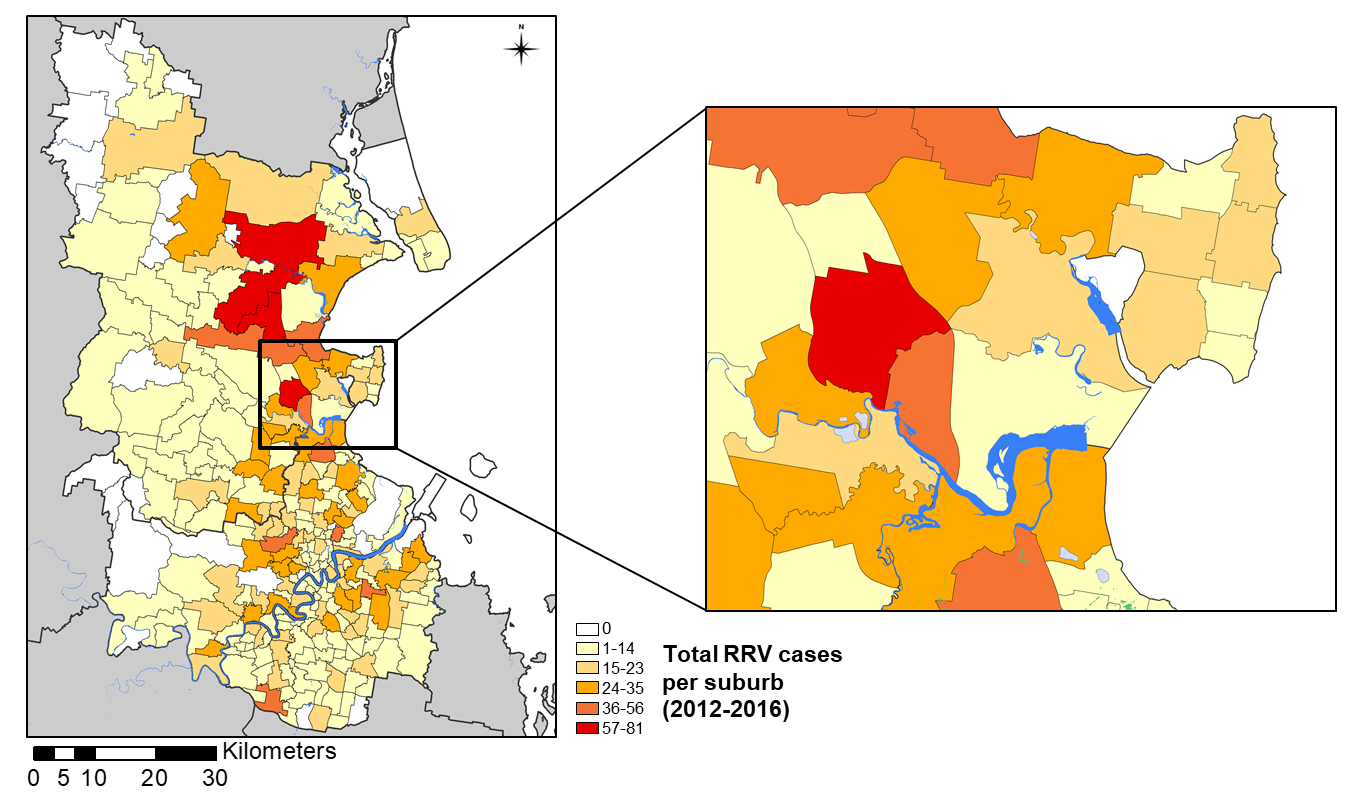


**Figure S1.** Human Ross River virus (RRV) cases for each suburb in the region of South East Queensland in which the study was performed for the period 2012-2016. Human RRV cases in the suburbs immediately surrounding the study area are shown in the map inset.
